# Research on the Performance of Superhydrophobic Cement-Based Materials Based on Composite Hydrophobic Agents

**DOI:** 10.3390/ma16196592

**Published:** 2023-10-07

**Authors:** Jie Luo, Yi Xu, Hongqiang Chu, Lu Yang, Zijian Song, Weizhun Jin, Xiaowen Wang, Yuan Xue

**Affiliations:** College of Mechanics and Materials, Hohai University, Nanjing 211100, China; luojiehhu@163.com (J.L.); chq782009@126.com (H.C.); yanglu90@hhu.edu.cn (L.Y.); songzijian@hhu.edu.cn (Z.S.); j_weizhun@163.com (W.J.); 18668911326@163.com (X.W.); 15038950772@163.com (Y.X.)

**Keywords:** compound hydrophobic agent, contact angle, mechanical properties, water absorption rate, pore structure

## Abstract

The utilization of a novel monolithic superhydrophobic cement material effectively prevents water infiltration and enhances the longevity of the material. A method for improving superhydrophobic concrete was investigated with the aim of increasing its strength and reducing its cost by compounding superhydrophobic substances with water repellents. The experimental tests encompassed the assessment of the compressive strength, contact angle, and water absorption of the superhydrophobic cementitious materials. The findings demonstrate that an increase in the dosage of isobutyltriethoxysilane (IBTES) progressively enhances the contact angle of the specimen, but significantly diminishes its compressive strength. The contact angle of SIKS mirrors that of SIS3, with a superior compressive strength that is 68% higher. Moreover, superhydrophobicity directly influences the water absorption of cementitious materials, with a more pronounced superhydrophobic effect leading to a lower water absorption rate. The water absorption of cementitious materials is influenced by the combined effect of porosity and superhydrophobicity. Furthermore, FT−IR tests unveil functional mappings, such as -CH_3_ which can reduce the surface energy of materials, signifying successful modification with hydrophobic substances.

## 1. Introduction

Within the field of engineering, the majority of structures are designed with a lifespan of at least 50 years [1]. Nevertheless, the innate hydrophilic properties of cementitious materials render them vulnerable to the ingress of corrosive agents utilizing water as a medium, consequently compromising their longevity. The hydrophilic characteristic inherent to cement-based materials frequently result in various challenges, including the accumulation of dust [2,3], deterioration due to freeze–thaw cycles [4,5], corrosion caused by chloride salts [6,7,8], sulfate-induced corrosion [9,10], and the corrosion of reinforcement [11,12]. Structures situated in specific environments, notably those characterized by high humidity, exhibit an elevated susceptibility to premature structural failure. Consequently, it is imperative to minimize the infiltration of moisture into these materials. Presently, researchers are directing their efforts toward the advancement of superhydrophobic surfaces that facilitate unhindered droplet runoff, preventing their adherence to the material’s surface [13]. This attribute serves as an effective deterrent against the infiltration of moisture into the inner regions of cementitious materials.

There are two methods for preparing superhydrophobic cement-based materials: surface modification [14,15,16] and monolithic modification [17]. Surface modification involves the application of coatings or impregnation to create a superhydrophobic film on the material’s surface. For example, Vanithakumari et al. [18] prepared superhydrophobic coatings using perfluoro octyl triethoxy silane and hexadecyl trimethoxy silane. Wang et al. [19] prepared superhydrophobic coatings by employing nonfluorinated triethoxyoctylsilane to reduce the material’s surface energy, while the combination of microscale diatomaceous earth and sand powders contributed to a hierarchical structure. Wang et al. [20] prepared a superhydrophobic coating using IBTES. However, it is important to note that superhydrophobic coatings are susceptible to environmental damage, which can significantly reduce their working lifespan. Superhydrophobic coatings can easily fail due to cracking of the concrete, aging and the peeling of coatings, etc. [21,22,23]. Therefore, when considering the long-term protection of concrete buildings, the limited duration of superhydrophobic coatings should be taken into account. 

Integral hydrophobic modification involves the use of different hydrophobic substances to lower the surface energy and various methods to create micro and nanostructures, thereby enhancing the material’s hydrophobicity. The integral hydrophobic modification of a material for superhydrophobicity can provide a comprehensive range of superhydrophobic properties, not just on the surface. Even if the surface of the material is damaged, the material will still retain its superhydrophobic properties internally, which can extend the effective working time of the material. Several researchers have successfully prepared superhydrophobic concrete using different hydrophobic substances [24,25,26]. Xu et al. [27] utilized stearic acid and paper sludge ash, ground into a hydrophobic powder with a contact angle of 153°. From an environmental perspective, this powder can replace some of the silicate cement. Wang et al. [28] prepared hydrophobic concrete by using stearic acid and further polishing the concrete surface with sandpaper, which results in superhydrophobic concrete with a contact angle of 153.7°.

Despite its potential advantages, it is vital to acknowledge that integral hydrophobic modification for achieving superhydrophobicity in materials may lead to a notable reduction in their mechanical properties. Wang et al. [29] conducted research demonstrating that superhydrophobic modification resulted in a substantial reduction of 30.9% in compressive strength and 18.1% in flexural strength compared with unmodified materials. Dong et al. [30] used oil-in-water suspension emulsions to prepare superhydrophobic concrete, and the compressive strength decreased significantly from 8.7 MPa to 1.4 MPa as the oil/water volume ratio increased from 1:1 to 4:1. Furthermore, it is worth noting that some of hydrophobic agents employed in these methodologies are environmentally unfriendly, containing polluting elements [18] such as fluorine (F). Additionally, these agents can significantly reduce the strength of cementitious materials, increase costs [31] and affect their practical application in engineering.

This study aims to prepare an environmentally friendly superhydrophobic cementitious material by utilizing a novel composite hydrophobic agent. The compound hydrophobic agent significantly improves the superhydrophobicity of the material, has a low effect on strength, and can reduce costs. When the contact angles are similar, the composite hydrophobic agent has a higher compressive strength and lower cost compared with IBTES. Additionally, this study investigates the influence of IBTES and the composite hydrophobic agent on the contact angle, mechanical properties and water absorption of the cementitious material. X-ray diffractometer (XRD) and Fourier transform infrared spectroscopy (FT−IR) are employed to explore the mechanisms underlying the superhydrophobic properties of the cementitious material, while mercury intrusion porosimetry (MIP) is used to analyze the pore structure of the superhydrophobic cementitious material.

## 2. Materials and Methods

### 2.1. Materials

Portland cement (P⋅II 42.5 compliant with GB 175-2007 and equivalent to CEM I 42.5) from Nanjing Hailu Cement Co., Ltd., Nanjing, China. was used. Nano silica (NS; BET: 200 m^2^/g, average diameter: 13 nm) was obtained from the Beimo company (Jiaxing, China). Silica fume (SF; Model 951) was obtained from the Elkem company, Oslo, Norway. Isobutyltriethoxysilane (IBTES) was obtained from RHΛWN, Shanghai, China. Polycarboxylic superplasticizer (water reducing efficiency: 30%) and waterproofing agent (model number: KLJ^®^-VI) were obtained from Sobute New Materials Co., Ltd., Nanjing, China.

### 2.2. Mix Proportion

The test reference ratio was a water/cement ratio (W/C) of 0.5 and a cement/sand ratio (C/S) of 1:2. The mix proportions are shown in Table 1. The dosages in the table are all mass fractions. All other substances were added as a percentage of the dosage of cement. SIS1, SIS2 and SIS3 groups were used to study the effect of different dosage of IBTES on cement-based materials. To reduce costs, the SIKS were considered compound hydrophobic agents. SIKS were doped with silane. For the comparison test, the SKS group contained only KLJ. PC1 and PC2 served as blank control groups for the study. In PC2, silica fume and silica nanoparticles were used as dopants, while PC1 remained free of any dosage agent.

### 2.3. Superhydrophobic Concrete Preparation

**Figure 1:**
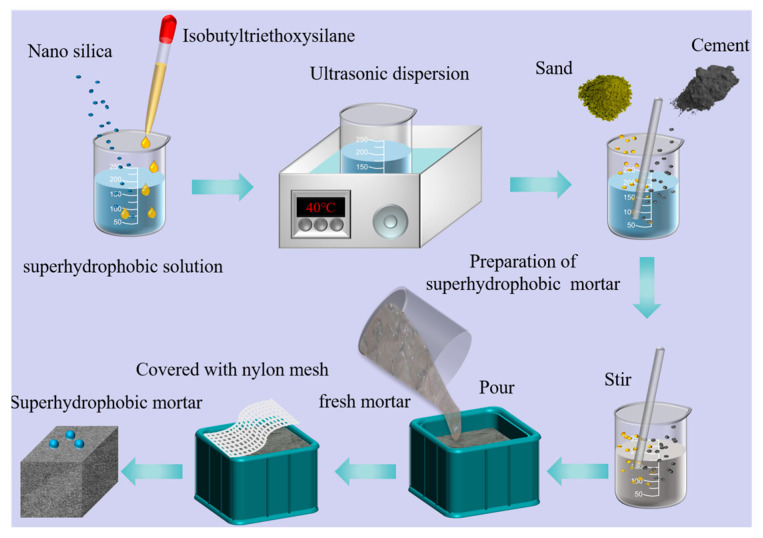
The schematic diagram of the fabrication process of superhydrophobic mortar.

The process of preparing superhydrophobic cement-based materials is shown in Figure 1. The mass of each raw material was weighed according to the test ratio, and the mixed solution was prepared by first dispersing the NS in water and stirring for 30 s. Then, the hydrophobic substance (IBTES, KLJ) was added to a coagulation solution of NS and water. The above solution was dispersed via ultrasonication at 40 °C for 30 min to prepare a superhydrophobic solution. When forming, cement, sand and SF were put into the mixer in turn. After stirring for 1 min, the super hydrophobic solution was then added and stirred for another 4 min to obtain fresh superhydrophobic mortar mixture. Then, the fresh superhydrophobic mortar mixture was poured into the mold, a nylon net was attached to the surface of the mold and the mold was demolded after 1 d and put into the maintenance room (temperature of 20 °C and humidity of 80%) for 28 d.

### 2.4. Test Methods

#### 2.4.1. Wettability Test

Contact angle (WCA) and contact angle hysteresis (CAH) were performed with a contact angle meter (DSA30, Kruess, Hamburg, Germany) at ambient temperature to characterize the superhydrophobic property. The volumes of probing liquids were approximately 5 μL for the contact angle measurement. Each contact angle reported was an average value of five independent measurements on different spots. The droplet images of the specimen surface were taken with the digital camera and macro lens that come with the contact angle measuring instrument.

#### 2.4.2. Compressive Strength Test

The compressive strength test was conducted in accordance with the Chinese national standard GB/T 17671-2021. All samples were cured for 28 d before testing. The cubic specimens (40 mm × 40 mm × 40 mm) were prepared to measure the compressive strength. The final result was determined by averaging the values obtained from three specimens.

#### 2.4.3. Waterproofing Ability Test

The water absorption test was performed with reference to the Chinese national specification JGJT 70-2009. The test was performed using repeated samples on ordinary and superhydrophobic cement-based materials, and after the age reached 28 d, these samples were put into an oven at 80 °C, dried to constant weight and then were put into water, and the distance from the surface of the specimen to the water surface was kept at 3 cm throughout the process. The weight of the specimen was weighed at regular intervals. The water absorption rate calculation formula is shown in Equation (1). Three specimens were taken from each group and the average value was taken after measurement, and the trend of the water absorption rate of each group was observed with time.

(1) $$W=\frac{m_{1}-m_{0}}{m_{0}}\times 100$$

where *W* is the water absorption of test pieces, *m*_1_ is the weight of test pieces at different times, and *m*_0_ is the initial weight of test pieces after drying.

#### 2.4.4. Characterization

The surface morphology of the samples was analyzed using a scanning electron microscope (SEM) model ZEISS Sigma 300, Carl Zeiss Microscopy GmbH, Oberkochen, Germany and their chemical compositions were investigated using energy-dispersive spectroscopy (EDS, ZEISS Sigma 300, Carl Zeiss Microscopy GmbH, Oberkochen, Germany). XRD analysis was used to characterize the specific composition of the sample product. The identification of phase compositions was conducted using an X-ray diffractometer (XRD, Rigaku-D/max 2200pc, Rigaku Corporation, Tokyo, Japan) with a Cu Ka (k = 1.54 Å) incident radiation. The 2θ scanning range was from 5° to 90° with a scanning speed of 2 °/min. The pore structure of the hardened cement paste was analyzed using mercury intrusion porosimetry (MIP, MicromeritiPC1 AutoPore V 9620, Micromeritics, Norcross, GA, USA). The analysis of functional groups was undertaken using Fourier transform infrared spectroscopy (FT−IR, Thermo Scientific Nicolet iS20, Thermo Fisher Scientific, Waltham, MA, USA).

## 3. Results and Discussion

### 3.1. Wettability

**Figure 2:**
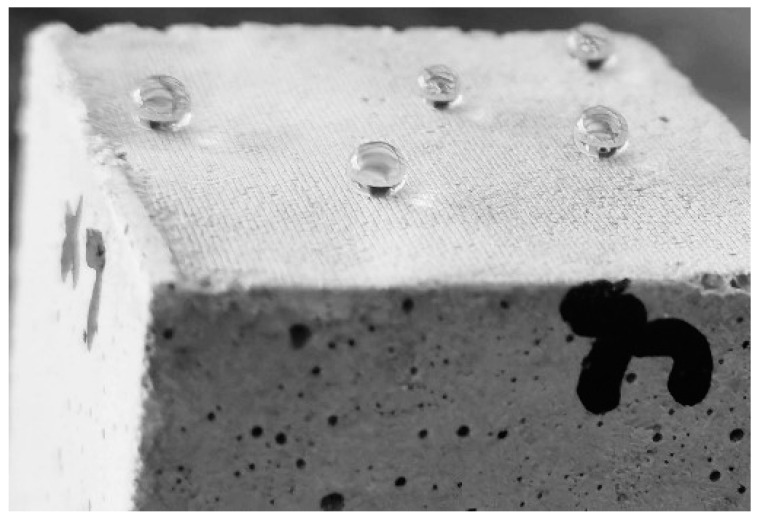
Morphology of the water droplet on superhydrophobic surfaces.

**Figure 3:**
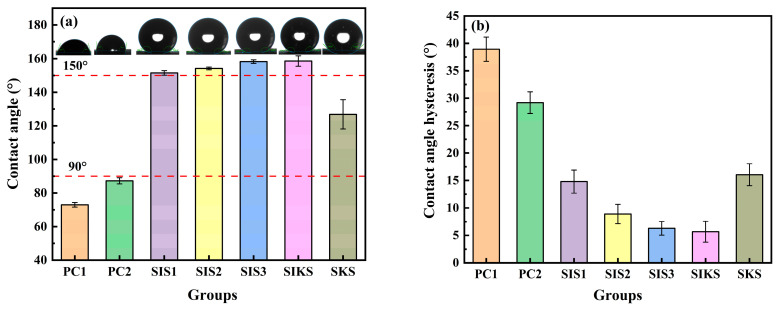
Effect of different hydrophobic agents on (**a**) contact angle and (**b**) contact angle hysteresis.

Figure 2 illustrates the morphology of the water droplets on a superhydrophobic surface. This indicates that the superhydrophobic surface was successfully prepared. The effect of the hydrophobic agent type and dosage on the contact angle is presented in Figure 3a. Within the single hydrophobic group (SIS1, SIS2, SIS3), evidence is presented that as the dosage of IBTES increases, the contact angle progressively rises, reaching a remarkable 158.3°. The plain concrete (PC1 and PC2) without hydrophobicity had a contact angle of less than 90°. The PC1 and PC2 exhibited hydrophilicity. The KLJ was able to modify the hydrophobicity of cement-based materials. But the contact angle of SKS was only 111.1°, and did not display superhydrophobicity. The composite hydrophobic agents prepared by using the KLJ and IBTES were effective in the superhydrophobic modification of cement-based materials. 

Upon comparing the composite hydrophobic agent with the single hydrophobic agent (SIKS compared with SIS1, SIS2, SIS3), it is evident that the IBTES dosage in the SIKS group was the same as that in SIS1, but the contact angle increased from 151.5° to 158.6° (Figure 3a). These results indicate that the addition of KLJ effectively enhanced the superhydrophobic modification when combined with IBTES. The superhydrophobicity of the SIKS and SIS3 were similar, with contact angles of approximately 158°. However, the SIS3 group contained double the amount of IBTES compared with the SIKS group, and this excessive IBTES content adversely affected the mechanical properties of cementitious materials. The contact angles of PC1 and PC2 were less than 90°, while the contact angles of the other groups were more than 90°. This indicates that hydrophobic substances can effectively convert the gelling material from hydrophilic to hydrophobic. The IBTES and KLJ reduced the surface energy of the material and modified the material. The contact angle hysteresis of SIS1, SIS2 and SIS3 decreased gradually (Figure 3b). PC1 and PC2 were not superhydrophobically modified, with a contact angle hysteresis of 29° or more. SIKS had a contact angle hysteresis of less than 10°. SKS was only hydrophobically modified and had a contact angle hysteresis of more than 10°.

**Figure 4:**
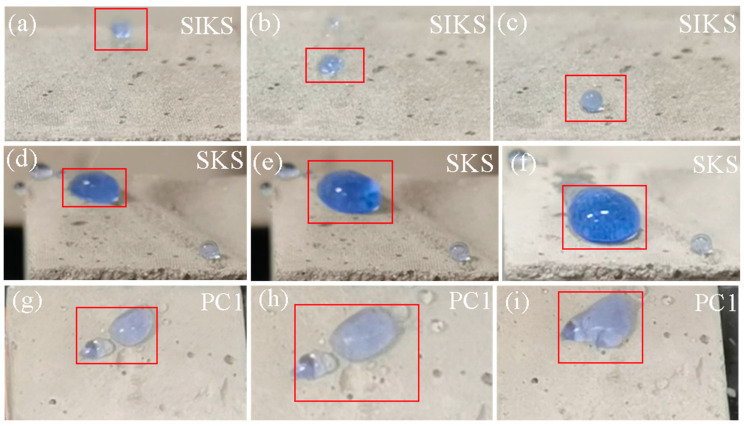
Water droplet wetting diagram on specimen surface: (**a**–**c**) superhydrophobic; (**d**–**f**) hydrophobic; (**g**–**i**) hydrophilic.

Figure 4 illustrates the wettability of various specimen surfaces. The superhydrophobic surface exhibited a water droplet in spherical form that swiftly rolled off, leaving no droplets behind. On the other hand, the hydrophobic surface showed a transition. As the water droplet increased in size, it gradually became oval and slowly slid off, usually leaving a mark on the surface. In contrast, the hydrophilic surface behaved differently. With an increasing volume of the water droplet, the surface gradually became wetted and did not facilitate the formation of a rolling water droplet.

**Figure 5:**
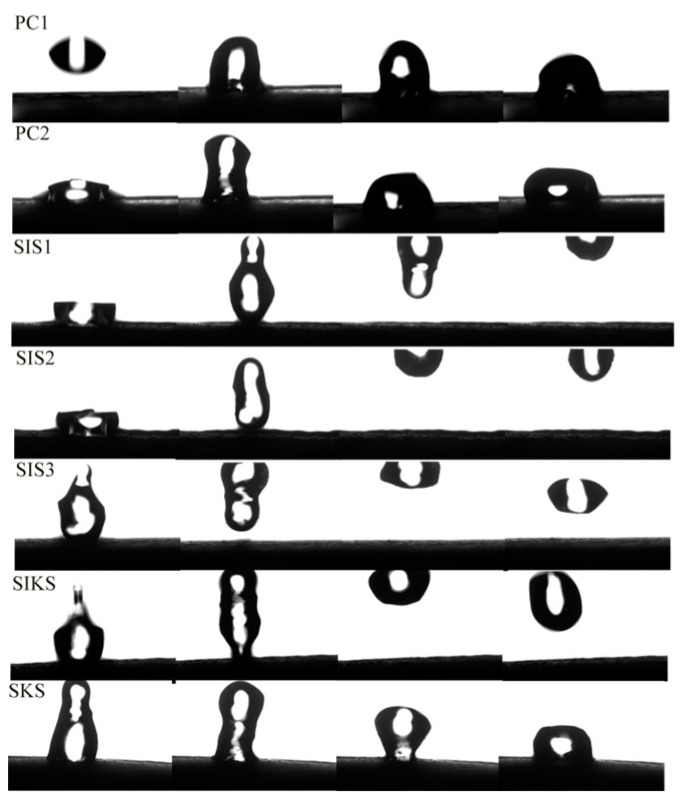
Dynamic bouncing processes of different groups.

**Figure 6:**
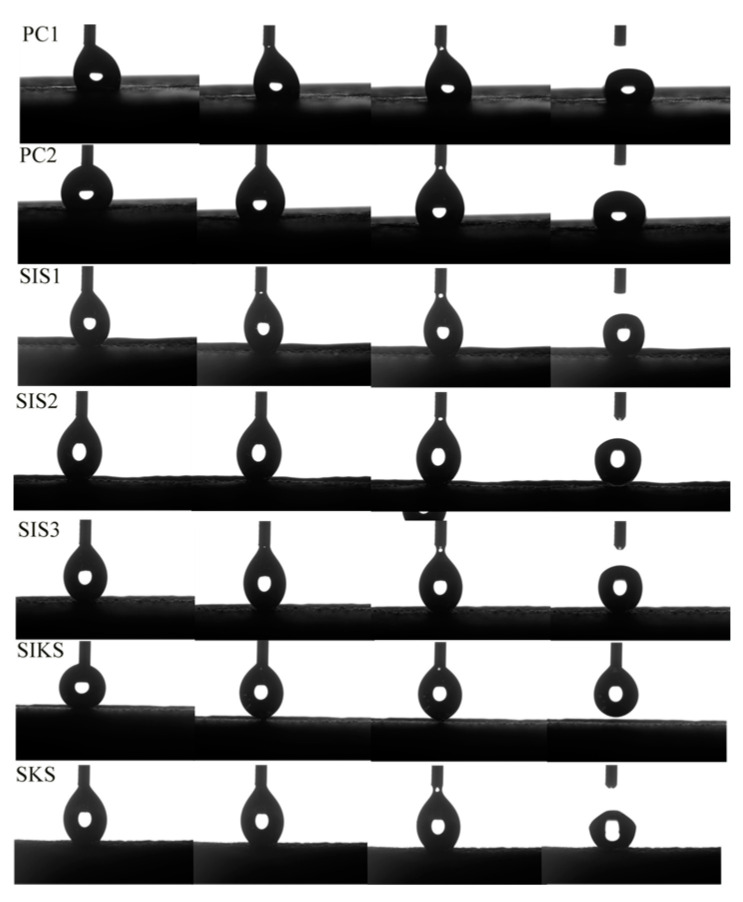
Water droplets touching different surfaces.

The dynamic bouncing processes of water droplets on the surface of different specimens are shown in Figure 5. A typical water bouncing process could be obtained on the surface of the superhydrophobic mortar, where the water droplets can leave the surface completely without leaving any residue. On PC1, PC2 and SKS surfaces, water droplets could not leave the surface. And water droplets were adsorbed by the surface. The SKS water bead bounce was higher than PC1 and PC2. This suggests that hydrophobic surfaces have an effect on dynamic bouncing process. When a water droplet touched the surface of PC1 and PC2, the droplet was adsorbed directly onto the surface and detached from the syringe (Figure 6). Water droplets were adsorbed on the surfaces of SIS1, SIS2 and SIS3 and were dislodged from the syringe. This suggests that the Cassie–Baxter state [32] is not stable enough. In SIKS, the water droplet did not adhere to the surface after contacting it. Finally, the water droplet adhered to the hydrophobic tip of the syringe and left the superhydrophobic surface when the syringe rose from the surface. This suggests that the Cassie–Baxter state is stable under external forces [33].

### 3.2. Compressive Strength

**Figure 7:**
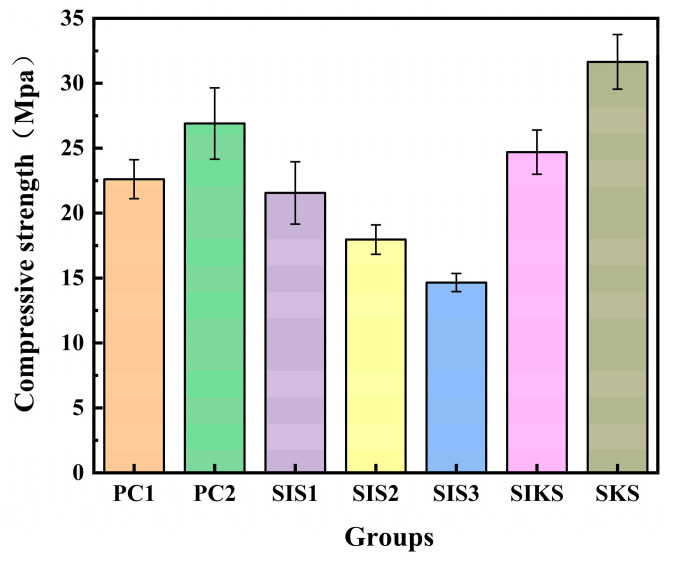
Effect of different hydrophobic agents on compressive strength.

The compressive properties of different hydrophobic agents on cement-based materials are depicted in Figure 7. The PC2 showed a higher compressive strength compared with the PC1 because PC2 is doped with nano-silica and silica fume. With an increase in IBTES dosage, the compressive strength of each SIS group gradually decreased. In comparison to the PC1, the addition of IBTES reduced the compressive strength of SIS1, SIS2 and SIS3 by 4.8%, 20.3% and 35.0%, respectively. Similarly, when contrasted with the PC2, the compressive strength of SIS1, SIS2 and SIS3 exhibited reductions of 21.9%, 33.1% and 45.4%, respectively. The compressive strength of the singly doped group declined sharply as the amount of IBTES increased, and when the contact angle reached 158° or higher the compressive strength decreased by more than 35%. In order to enhance the compressive strength without affecting the superhydrophobicity, a compound hydrophobic agent was used instead of a single hydrophobic agent. Compared with PC1, the compressive strength of SIKS improved by 9.3%. The compound hydrophobic agent could improve the compressive strength while maintaining a contact angle above 155°. Compared with SIS3, the compressive strength of SIKS increased by 68.0%. The contact angles of SIS3 and SIKS were close, but the compressive strength of SIKS was higher than that of SIS3. Although the compressive strength of SKS was higher than that of SIKS, the contact angle of SIKS was lower than 150°, which was not superhydrophobic. This suggests that the effect of IBTES on superhydrophobicity was more important. The KLJ could only hydrophobically modify mortar, but had a positive effect on strength.

The KLJ exhibited greater strength compared with both PC1 and PC2 due to its ability to react with cement hydration products, forming insoluble crystals that block concrete capillaries and enhance concrete density [34]. The KLJ also refined the capillary structure of concrete, increased its density, and mitigated the adverse effects of IBTES on strength. The SIKS was enriched with nanomaterials, yielding a positive effect on strength enhancement [35]. Meanwhile, the substitution of a portion of IBTES with KLJ further bolstered the material’s strength. Nanoparticles and silica fume [36] facilitated cement hydration, consequently enhancing the material’s compressive strength while mitigating the detrimental effects of hydrophobic agents. Nano-silica and silica fume possessed fine particles capable of filling mortar pores and micro-cracks, thereby increasing the mortar compactness and density. Additionally, nano-silica and silica fume can serve as crystalline nuclei, fostering hydration reactions during cement gel formation [37]. The hydrophobic substance affected cement hydration and reduced the strength of mortar. The IBTES does not contain element F and is environmentally friendly, but is expensive. The KLJ replaced some of the IBTES and can effectively reduce costs. While the contact angle of SIKS closely resembles that of SIS3, its compressive strength significantly surpassed that of SIS3. This observation implies that the composite hydrophobic agent can achieve the superhydrophobic modification of cementitious material with minimal effects on compressive strength.

### 3.3. Water Absorption Rate

**Figure 8:**
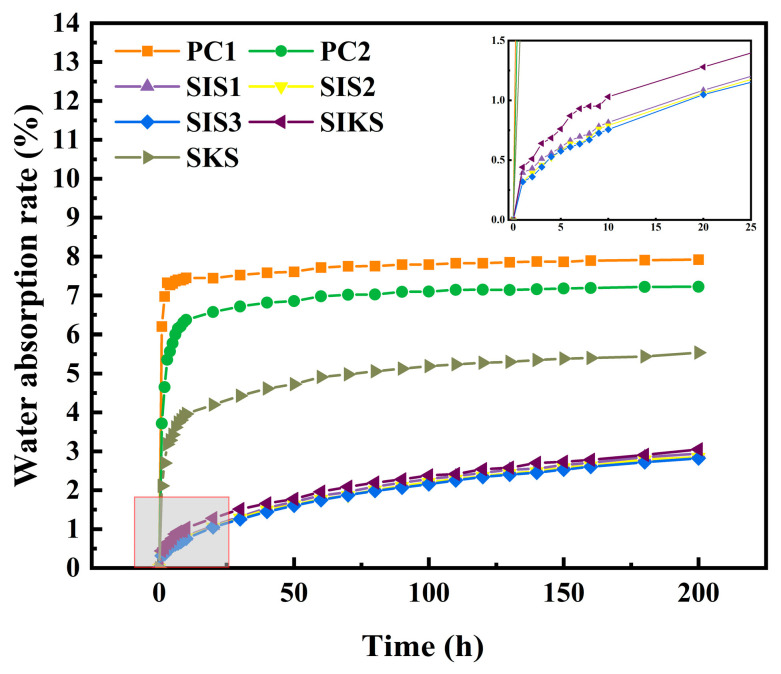
Variation of water absorption rate of each group with time.

The water absorption rates of the specimens in different groups are shown in Figure 8, and the water absorption rates of the other superhydrophobic test groups were lower compared with PC1 and PC2. Initially, the water absorption rates rose rapidly in all groups. As the water absorption of the specimen reached saturation, the rate of increase of water absorption gradually slowed down and eventually stabilized at a constant value. In the first hour, the PC1 group had a water absorption rate of 6.2%. The water absorption of the PC2 with nanomaterials was much lower, at 3.7%, a reduction of about 40%. After 200 h, PC1 and PC2 had water absorptions of 7.9% and 7.2%, respectively. In the single dosage group (SIS1, SIS2, SIS3), as the amount of IBTES increased, superhydrophobicity gradually increased and the water absorption decreased significantly. In the first hour, the highest water absorption rate among these groups was only 0.4%, much lower than PC1 and PC2. Following 200 h, the water absorption rates in the single-doped groups (SIS1, SIS2, SIS3) were 2.9%, 2.9% and 2.8%, respectively. These values signify a noteworthy decline of 62.9%, 64.0% and 64.4% in comparison with PC1. Superhydrophobic cement-based materials effectively hindered moisture from infiltrating the material’s interior, which resulted in a decrease in water absorption rates as superhydrophobicity increased. The SKS exhibited a 5.5% water absorption rate after 200 h, marking a reduction of 29.9% compared with PC1. The SKS had a contact angle of less than 150°, which was not superhydrophobic and offered limited waterproofing protection inside the material. As a result, it had a higher water absorption rate than the other superhydrophobic groups. In contrast, the compound hydrophobic agent (SIKS) yielded promising outcomes and the water absorption rate was consistently below 1% within the first hour. Over 200 h, the water absorption rate for SIKS remained below 4%, indicating a substantial 59.3% reduction compared with PC1.

The water absorption rate of PC2 is lower than that of PC1. This difference is due to the incorporation of nanomaterials in the PC2, which results in material compaction and a reduction in water absorption. This suggests that filling the pores of the cementitious material with nanomaterials has a certain effect on reducing water absorption, although it is not very pronounced. The reduction in water absorption can be attributed to two factors: the improvement in material densification [38] and the hydrophobic modification of the material [39]. The water absorption of the test group mixed with hydrophobic material was significantly lower, at only 4.4%, while the water absorption of the PC1 group was 7.9%. Achieving a significant reduction in water absorption and improving material durability required the transformation of hydrophilic materials into superhydrophobic ones. This transformation fundamentally prevented water infiltration into the material. The contact angle of the SKS was below 150°. This suggests that the single-doped hydrophobic chemical pore bolus had a lesser effect on material modification when compared with other test groups. As a result, the water absorption rate in the SKS group exceeded that of other superhydrophobic test groups. This demonstrated a clear correlation between the superhydrophobic effectiveness of modification and water absorption. Enhanced superhydrophobicity led to a decreased rate of water infiltration into the material, which resulted in reduced water absorption.

### 3.4. SEM Analysis

**Figure 9:**
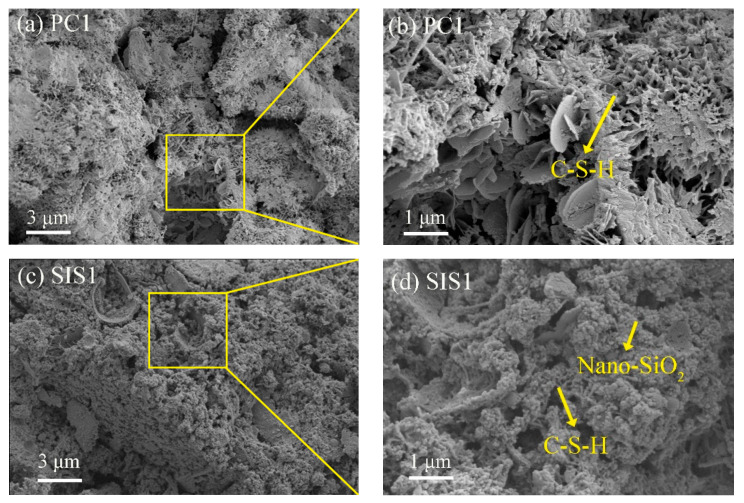

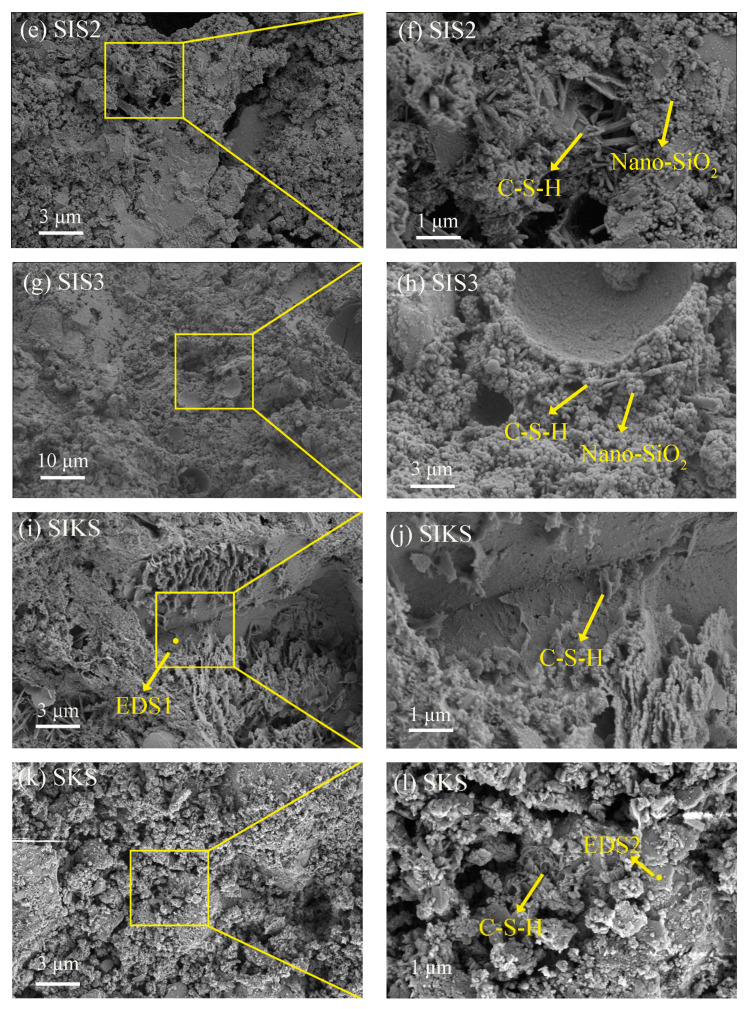
Micro morphology of different groups.

**Figure 10:**
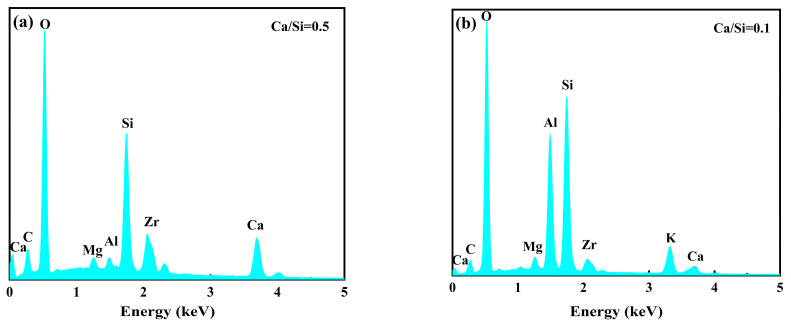
EDS spectrum of (**a**) SIKS and (**b**) SKS.

Figure 9 shows the SEM images of the superhydrophobic concrete at different magnifications. At the 3 µm scale, the structure of PC1 was looser, with many pores and C-S-H cross-linking each other (Figure 9a). When the magnification was 30 KX, the microscopic morphology of the PC1 surface was that of inter-crosslinked C-S-H. Flaky C-S-H gels were generated in PC1, which indicates a high degree of hydration. The morphologies of SIS1, SIS2 and SIS3 are relatively similar, all being C-S-H gels crosslinked with each other and featuring numerous nano-silica particles on their surfaces. These silica nanoparticles created rough structures that significantly enhanced the superhydrophobicity of cement-based materials. Roughness plays a crucial role in the development of superhydrophobic surfaces. The internal structure of the superhydrophobic specimen revealed the presence of numerous micro- and nano-scale bumps. These rough structures facilitated the capture and retention of air layers, allowing the water droplet to form large contact angles and effortlessly roll off inclined surfaces [40,41]. As the amount of IBTES dosage increased, there was a gradual increase in the number of micro-nano structures within the material. The micro-nano structures further enhanced the superhydrophobic modification effect. The SIS1 exhibited lower porosity, the SIS2 formed a lamellar C-S-H gel with a higher degree of hydration, and the SIS3 possessed a looser structure. These characteristics aligned with their respective compressive strength results. With the increase in IBTES dosage, the structure gradually became looser, and the pore space gradually expanded. The presence of hydrophobic substances in the cement paste had a notable effect. The hydrophobic substance reduced the fluidity of the cement paste, which made it challenging for water to permeate between the cement particles and hydrophobic substances and affected the hydration reaction of the cement. The SIKS produced a denser structure of layered C-S-H gels (Figure 9i). This was consistent with its higher compressive strength results. The amount of IBTES in SIKS was smaller, so the structure of the SIKS was denser compared with SIS3. This is consistent with the previous results of compressive strength. The SKS has many fine particles within it, which fills the pores and makes the structure denser. Figure 10 shows the elemental analysis of SIKS (Figure 9i, EDS1) and SKS (Figure 9l, EDS2). The SIKS and SKS contained basically the same elements, mainly elements contained in the hydration products of cement, such as Ca, Si, etc. The Ca/Si of SIKS was about 0.5, within the typical range of C–S–H gel [42]. The Ca/Si of SIKS was about 0.1. This shows that it is not a C-S-H gel. The C content of SIKS was higher than the SKS. This suggests that there was more hydrophobic material in the SIKS hydration product.

### 3.5. XRD Analysis

**Figure 11:**
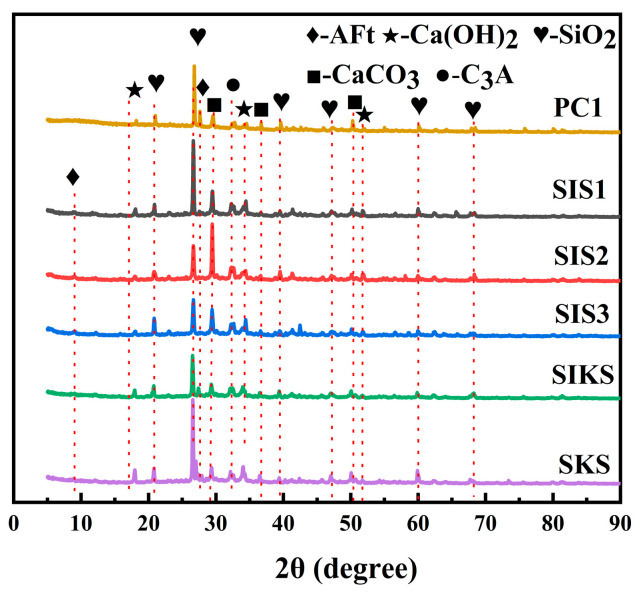
XRD patterns of superhydrophobic concrete prepared with different hydrophobic agent types.

XRD is used to analyze the specific chemical composition of the material. The crystal structures of the different specimens were detected using the XRD technique to determine their main components. The main components of different specimens were similar, both containing Ca(OH)_2_, AFt, SiO_2_, etc. (Figure 11). This indicates that IBTES and KLJ did not change the chemical composition of the specimens. The highest peak in the position of 26.6° was in accordance with quartz sand (JCPDS No. 46–1045). Peaks at 18.0° and 34.2° were in line with calcium hydroxide (JCPDS No. 04–0733) and the peak at 9.0° was in keeping with AFt (JCPDS No. 41–1451). This was similar to the results of reference [43]. The peaks of Ca(OH)_2_ in SKS were much higher than the other groups, indicating that IBTES affected the hydration of cement to produce C-S-H gels. Different hydrophobic substances do not produce new peaks, which indicates that hydrophobic substances do not contribute much to the crystal structure [44]. 

### 3.6. FT−IR Analysis

**Figure 12:**
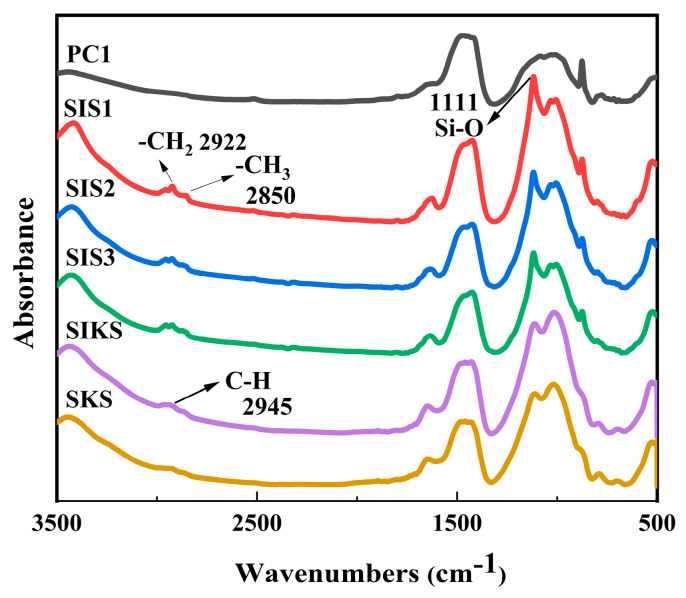
FT−IR patterns of superhydrophobic concrete prepared with different hydrophobic agent types.

In addition to the surface morphology, the chemical composition also affects the wettability of the material surface. In order to characterize the effect of superhydrophobic modification, an analysis of functional groups was performed using FT−IR. Figure 12 shows the FT−IR spectra of the different specimens. A stretching vibration peak of -OH of water was present at 3437 cm^−1^ for each group. In superhydrophobic concrete, the peak observed at 2945 cm^−1^ may be the stretching vibration of C-H in hydrophobic substances. Distinct peaks were observed near 2922 cm^−1^ and 2850 cm^−1^ in the SIS1, which confirms the presence of -CH_2_ and -CH_3_ functional groups. The stretching vibration of Si-O was observed at 1111 cm^−1^ for SIS1, SIS2, SIS3, SIKS and SKS. The presence of hydrophobic functional groups within the material was confirmed by the observed absorption peaks. However, PC1 did not show hydrophobic functional groups at the same position. These substances were effectively integrated into the cementitious materials through a sequence of chemical processes involving hydrolysis, dehydration and condensation [45], which resulted in the development of superhydrophobic properties within the cementitious materials.

**Figure 13:**
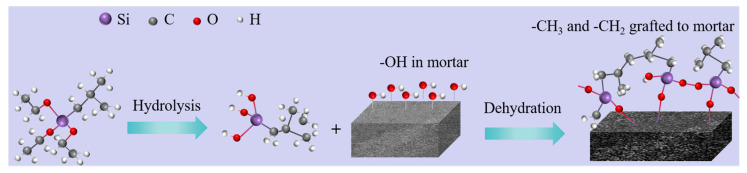
Superhydrophobic modification mechanism of hydrophobic agents.

The mechanism of superhydrophobicity is depicted in Figure 13. The siloxane group [46] undergoes hydrolysis to form a silanol group. The silanol group readily undergoes a dehydration condensation reaction with the hydroxyl group. This process facilitates the adhesion of low surface energy substances to the material’s surface, effectively reducing the solid surface energy. According to the Cassie–Baxter theoretical model [32], an increase in solid surface roughness leads to an increase in the contact angle on a hydrophobic surface. Merely reducing the surface energy of solids can only modify hydrophilic materials to hydrophobic materials; it cannot achieve a superhydrophobic effect. Therefore, a rough structure was introduced on the material surface using a nylon net. This dual action of low surface energy and rough structure modified the material into a superhydrophobic state. The SKS group had a higher compressive strength than the other test groups, and its contact angle was also greater than 90°. This indicates that the KLJ not only reduced the surface energy of the material, but also promoted the hydration of the cement and increased the compressive strength of the material. Composite hydrophobic agents prepared from KLJ and IBTES had a better effect on superhydrophobicity and compressive strength. The spectral analysis of SIKS and SKS contained C (Figure 10). Spectral analysis by SIKS shows that the C-S-H gels contained C. This suggests that IBTES hydrolyzed and cross-linked with the C-S-H gels. 

### 3.7. MIP Analysis

**Figure 14:**
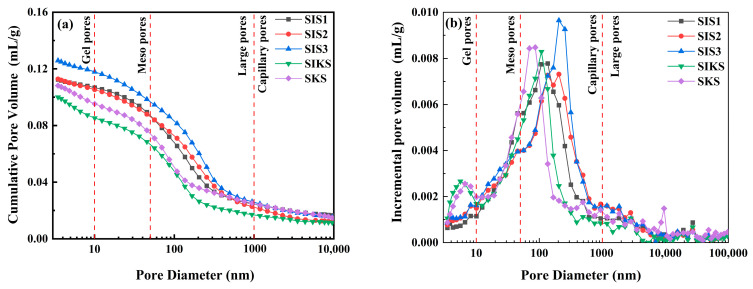

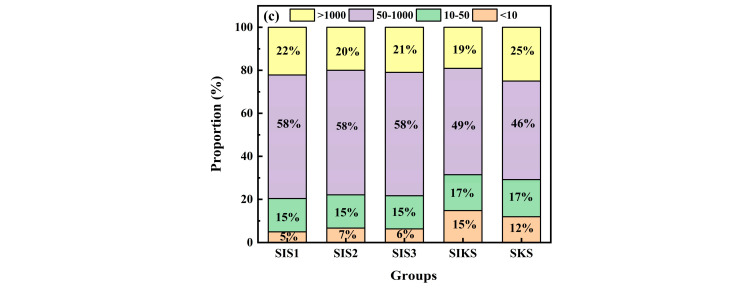
MIP test for pore size distribution: (**a**) cumulative pore volume; (**b**) pore structure distribution; (**c**) pore diameter distribution.

The pore parameters of the superhydrophobic cementitious materials prepared with different hydrophobic agents are shown in Figure 14. The pore volumes of the SIS1, SIS2, SIS3, SIKS and SKS groups were 0.1124, 0.1129, 0.1258, 0.1000 and 0.1081 mL/g, respectively (Figure 14a). The pore volume in the compound dosage group (SIKS) was lower than that in the single dosage group (SIS1, SIS2, SIS3). This suggests that the KLJ contributes to pore compaction and structural densification. The pore volume steadily rose within the single dosage group (SIS1, SIS2, SIS3) as the IBTES dosage increased. This phenomenon may be attributed to the influence of IBTES on hydration, impeding the compact growth of C-S-H. The difference in pore volumes between the groups was not significant and indicated that the nanomaterials filled the pores and enhanced material compactness. Mercury compression measurements show that the SIKS and SKS groups had the lowest porosity, while the SIS3 group exhibited the lowest water absorption. This indicates that porosity had an impact on water absorption, but it is important to recognize that superhydrophobicity also exerts an influence on water absorption. The porosity and superhydrophobicity play a combined role in determining water absorption in cementitious materials.

Related studies have shown that pores with a pore size below 10 nm are gel pores, capillary pores with a pore size between 10 and 50 nm are mesopores, and pores with a pore size between 50 and 10,000 nm are referred to as macropores [47]. It is generally believed that the more pores less than 50 nm, and the fewer the pores larger than 100 nm, the better the performance of concrete. The pore distribution maps of different specimens demonstrated percentages of different pore sizes by 10 nm, 50 nm and 1000 nm (Figure 14b). The percentages of macropores in the SIS1, SIS2, SIS3, SIKS and SKS groups were 79.61%, 77.92%, 78.32%, 68.56% and 70.83%, respectively. The total porosity in the SIS1, SIS2, SIS, SIKS and SKS groups are shown in Table 2. The larger the percentage of large pores, the more unfavorable it is for cementitious materials. Nano-silica and silica fume can densify the pore space, but the hydrophobic agent will be wrapped on the surface of cement particles and affect the hydration of cement. The hydrophobic agent affects the gelling of hydration products and hinders the connection of cement particles to form a continuous network structure. This, in turn, causes loose hydration products to impact the pore space within the structure. The volcanic ash effect generates more C-S-H gels, which leads to an increase in the number of mesopores and gel pores in the cementite. Another important pore parameter is the threshold pore size, which is closely related to the connectivity of the pores and the permeability of the cementitious material. The threshold apertures for the SIS1, SIS2, SIS3, SIKS and SKS groups are shown in Table 2. The SKS had the smallest threshold apertures. This indicates that the pore connectivity of the SKS group was lower and the pore structure was denser [48,49]. This was consistent with the results of the SKS micro-morphology. The SEM picture of SKS had a dense structure with a smaller pore structure (Figure 9k).

## 4. Conclusions

The effects of the composite hydrophobic agent and IBTES on the contact angle, mechanical properties and water absorption of cementitious materials were investigated. The mechanism related to the superhydrophobic properties of cementitious materials was investigated using XRD, FT−IR and other microscopic tests, and the pore structure of superhydrophobic cementitious materials was studied using MIP, and the following conclusions can be drawn:

(1) With the increase in IBTES dosage, the superhydrophobicity of the specimen gradually increased, but the strength decreased significantly. The compressive strength of composite hydrophobic agent was higher than that of IBTES when the contact angle was close, and the cost was lower. By using the lower-cost KLJ instead of higher-cost IBTES, the cost can be reduced and engineering applications will be facilitated. Hydrophobic substances have a detrimental effect on the compressive strength of cementitious materials. In the SKS group, the superior effect of compressive strength is attributed to the promotion of the hydration reaction by the nanomaterials, which outweighs the inhibition of the hydration reaction by the hydrophobic substances.
(2) The water absorption rates of SIS1, SIS2, SIS3 and SIKS groups after 200 h were less than 3.1%. Compared with PC1, the water absorption rates were reduced by more than 59%. The water absorption is related to two factors: porosity and superhydrophobicity. The effect of superhydrophobic modification is directly related to the water absorption rate. The better the modification effect, the more difficult it is for water to enter the interior of the material, and the lower the water absorption rate of the material.
(3) FT−IR analysis showed that hydrophobic functional groups were successfully grafted onto the material. The incorporation of IBTES increased the porosity of the material, while the LJK facilitated the dense structural pores and reduced the porosity.

## Figures and Tables

**Table 1:** Matching ratio design (mass fraction %).

Sample	Cement/g	W/C	C/S	NS	SF	IBTES	KLJ
PC1	600	0.5	0.5	-	-	-	-
PC2	600	0.5	0.5	2%	6%	-	-
SIS1	600	0.5	0.5	2%	6%	4%	-
SIS2	600	0.5	0.5	2%	6%	6%	-
SIS3	600	0.5	0.5	2%	6%	8%	-
SIKS	600	0.5	0.5	2%	6%	4%	8%
SKS	600	0.5	0.5	2%	6%		8%

**Table 2:** Total porosity and the most probable pore diameter for different samples.

	SIS1	SIS2	SIS3	SIKS	SKS
Total porosity (%)	21.59	21.58	23.33	19.14	20.99
The most probable pore diameter (nm)	136.14	205.17	205.08	108.22	86.37

## Data Availability

Not applicable.
